# Atypical coronoid process displacement in ZMC Trauma: Technical considerations and management algorithm – A technical note

**DOI:** 10.1016/j.jobcr.2025.05.003

**Published:** 2025-05-19

**Authors:** Jishnu Mohan, Virendra Singh, Ankita Dahiya, Rajesh Chauhan, Rashmi Bawane

**Affiliations:** Department of Oral and Maxillofacial Surgery, Post Graduate Institute of Dental Sciences, Rohtak, 124001, Haryana, India

## 1 Introduction

**Fig. 1:**
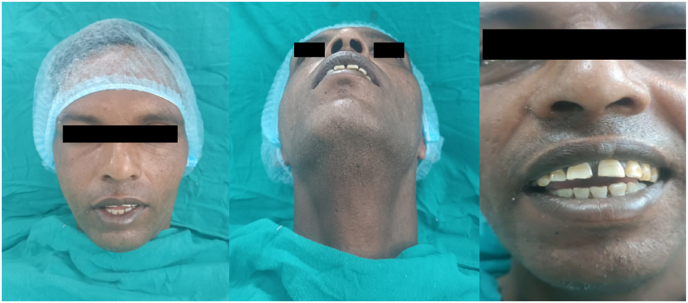
Preoperative photograph of the patient's face showing depression of the affected cheek (malar flattening) and an inability to fully close the mouth (open bite).

**Fig. 2:**
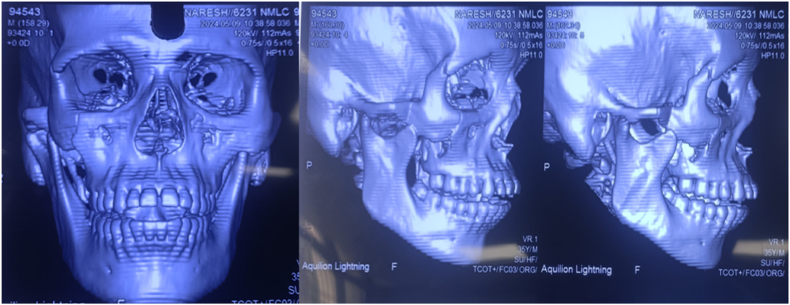
Preoperative CT scan (coronal or 3D view) showing the displaced coronoid process (arrow) positioned anterior to the fractured zygomatic arch segment. This confirms the mechanical locking of the mandible.

**Fig. 3:**
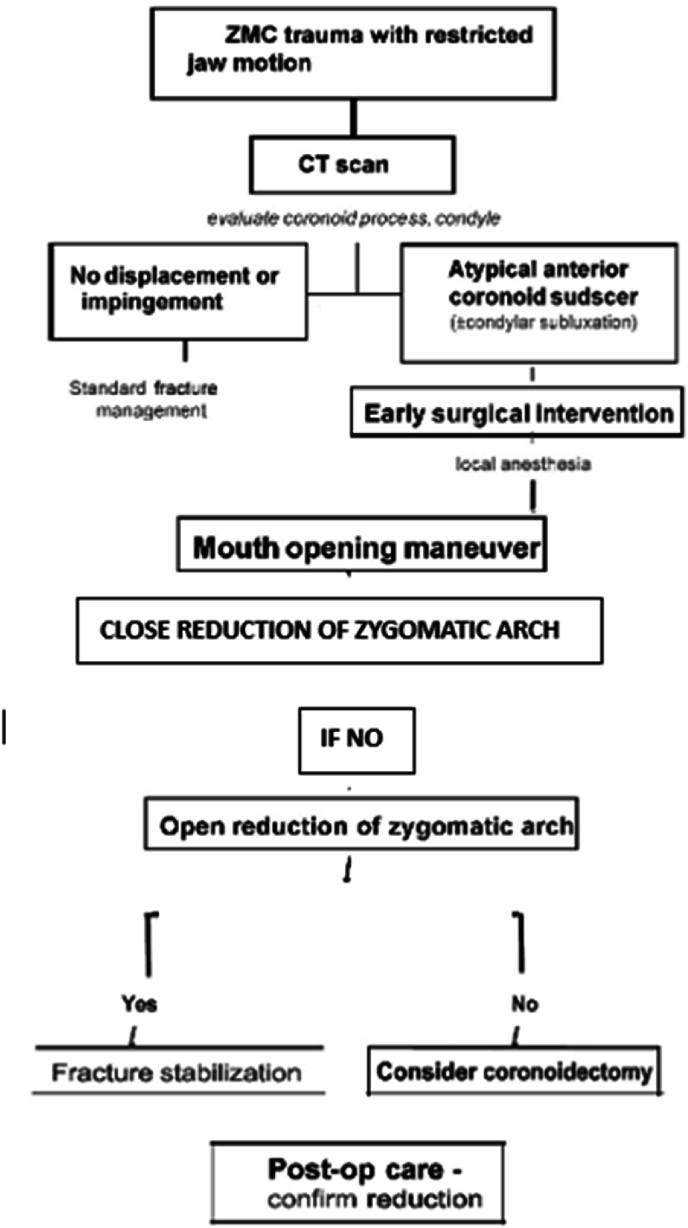
Flowchart illustrating a proposed management algorithm for atypical coronoid process displacement associated with ZMC fractures. The algorithm covers initial evaluation, imaging, decision making, surgical steps, and postoperative care in sequence.

**Fig. 4:**
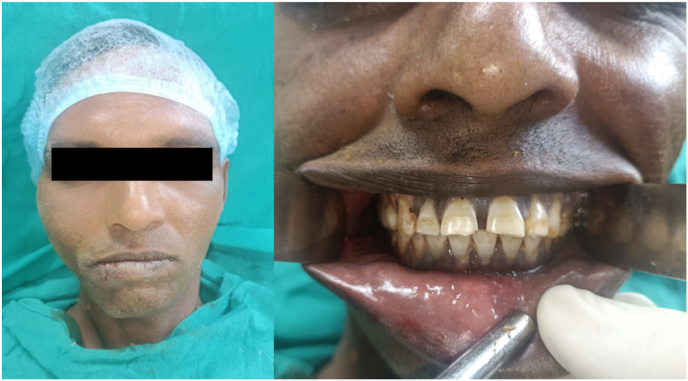
Postoperative CT scan (or panoramic X-ray) confirming anatomic reduction of the zygomatic arch (arrow) with the coronoid process returned to its normal position beneath the arch.

**Fig. 5:**
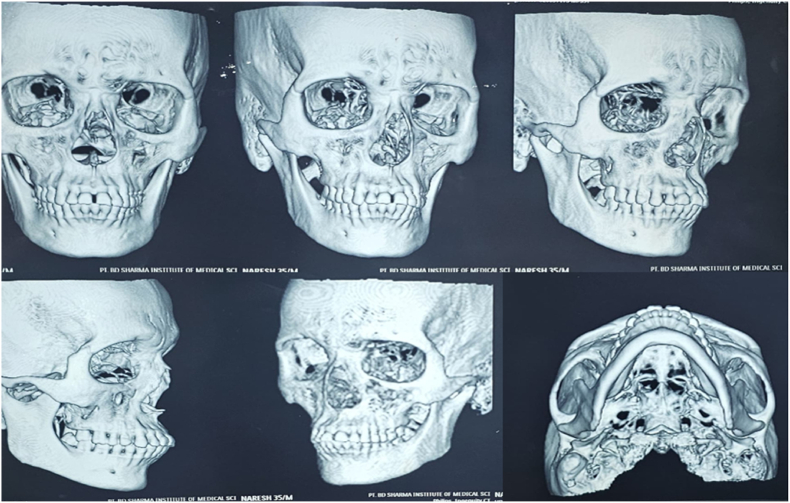
Postoperative photograph of the patient demonstrating improved facial symmetry and the ability to achieve normal occlussion.

Zygomaticomaxillary complex (ZMC) fractures, resulting from blunt force trauma to the periorbital area, are the second most common facial fractures after nasal fractures.^1^ These tetrapod malar fractures can be managed through various approaches, ranging from closed treatment to four-point open reduction and internal fixation (ORIF). While fractures of the mandibular coronoid process often accompany zygomatic fractures, no cases of coronoid dislocation are very rare.^2^ This unique case presents an anterior dislocation of the coronoid process over a ZMC fracture, along with the surgical technique used to manage it. Coronoid impingement in ZMC fractures can occur when the zygomatic arch is displaced medially, obstructing the coronoid process's normal movement. Patients may experience trismus, pain during mandibular movements, and difficulty with lateral excursions. CT scans with 3D reconstruction are essential for accurate diagnosis. Treatment options vary from reduction of the ZMC fracture to coronoidectomy in severe cases.^3^ The surgical approach may be intraoral or extraoral, depending on the fracture pattern and surgeon's preference. Post-operative care emphasizes early mobilization and physiotherapy to maintain range of motion and prevent re-ankylosis. Long-term follow-up is crucial to monitor for complications such as re-displacement, malocclusion, or persistent trismus. Understanding the biomechanical relationship between the zygomatic complex and coronoid process is vital for optimal surgical planning and outcomes in these complex facial fractures^2^ (see Fig. 1, Fig. 2, Fig. 5, Fig. 3, Fig. 4).

## 3 Discussion

As an OMFS team in apex trauma center, we encountered an unprecedented case: a complex ZMC fracture so severe that it prevented the coronoid process from returning to its original position. This unique case raises questions about the injury mechanism, contributing factors, reduction risks, and potential complications.

The literature review revealed no similar cases. While zygomatic fractures with coronoid process impingement or fractures are common, an anterior coronoid process dislocation related to a zygomatic fracture is exceptional.^3^

Several theories attempt to explain this phenomenon.

1. Open mouth during fall: This could explain the dislocation without fracturing the coronoid process, but questions arise about potential condyle subluxation.
2. Lateral impact causing condyle fracture and dislocation: However, no mandibular process fractures were observed.
3. Concentrated impact on the cheek with open mouth: This may have displaced the coronoid process, locking it anterior to the fracture upon mouth closure, subsequently dislocating the condyle.

Treatment approaches for such cases vary. Manual manipulation is often preferred for condylar dislocation reduction, while coronoid process fractures may be managed conservatively or surgically, depending on severity.

The unfractured state of the coronoid process, despite its dislocation, aligns with previous observations attributing coronoid fractures to severe temporalis muscle contraction rather than direct trauma.

This case suggests that coronoid process dislocation led to lateral condylar process dislocation, not vice versa. The temporalis muscle was likely under significant tension, raising concerns about potential injury during reduction.^8^

CT scans proved crucial in diagnosing this rare condition. Early treatment and physiotherapy are vital to prevent complications such as fibro-osseous ankylosis and to maintain mouth opening range. This case underscores the importance of considering atypical injury patterns^9^ in complex facial traumas and highlights the value of advanced imaging in diagnosis and treatment planning.

## 4 Conclusion

Atypical anterior displacement of the mandibular coronoid process in the setting of ZMC fracture is an exceedingly uncommon but clinically significant entity. It should be suspected in any patient with a zygomatic complex injury who presents with marked trismus or an inability to occlude properly, especially when standard fracture patterns do not explain the degree of jaw restriction. Prompt imaging (CT scan) is crucial to diagnose this condition. The cornerstone of management is timely surgical intervention to unlock the jaw by reducing the zygomatic arch fracture – facilitated by intraoperative maximal mouth opening maneuvers – which allows the coronoid process to return to its anatomical position. Rigid fixation of the ZMC, when indicated, helps prevent relapse of the displacement. Our proposed management algorithm provides clinicians with a step-by-step guide, from initial assessment through postoperative care, for handling this unusual injury. With appropriate management, patients can achieve excellent functional recovery. Early physiotherapy and follow-up are important to ensure long-term normal mandibular function and to monitor for any complications such as TMJ ankylosis or heterotopic bone formation. This case and review serve to fill a gap in the literature by describing the clinical presentation, surgical technique, biomechanical considerations, and an algorithmic approach to an atypical coronoid process displacement in ZMC trauma.

## 2 Case presentation and surgical techinique

A 36-year-old male patient reported to the Department of Oral and Maxillofacial Surgery with the chief complaint of inability to either open or close his mouth completely following a fall. The patient had slipped and experienced direct blunt trauma to the right cheek region, immediately after which he experienced difficulty in chewing.

Examination revealed the patient had bilateral end-on molar relation on the second molars with associated posterior open bite in the molar-premolar region and an anterior open bite. The patient was unable to open his mouth completely when asked to do so.

General examination showed the patient was conscious and well-oriented. The maxillofacial evaluation revealed subconjunctival hemorrhage and depression of the right malar eminence, with tenderness upon palpation at four sites of suture articulation. The ophthalmological exam was normal with no evident ocular signs.

Computed tomography (CT) of the facial bones confirmed a right ZMC fracture with an atypical anterior displacement of the right mandibular coronoid process without fracture (the coronoid lodged anterior to the fractured zygomatic arch segment). The ipsilateral condyle was noted to be subluxated anterolaterally out of the glenoid fossa on the CT scan (i.e. partial temporomandibular joint (TMJ) dislocation). The unusual coronoid position effectively “locked” the mandible, explaining the patient's inability to close his jaws. No other facial fractures were present. Based on these findings, a plan was made for urgent operative intervention after initial management of pain and facial soft tissue edema.

The patient was planned for zygomatic arch elevation using an intraoral Keen's approach with a modification in the technique under local anesthesia. As the fracture of the zygomaticomaxillary complex (ZMC) was rotated on the vertical axis, it was expected to be stable after reduction.^2^^,^^4^

Eleven days after trauma, the patient underwent reduction under local anesthesia. The technical modification included achieving mouth opening with a mouth gag or mechanical opening after anesthesia, followed by the insertion of a Bristow elevator posteromedially along the buttress beneath the arch. The patient was asked to keep his mouth open as much as possible, assisted by the mouth gag. The arch was then lifted with the Bristow elevator. The projection of the arch was achieved after the characteristic "pop" sound indicating reduction. The patient was then asked to close his mouth, which resulted in proper occlusion, indicating the clinical sign of appropriate fracture reduction.

The intraoral incision was sutured with 3-0 Vicryl. Immediate post-op evaluation showed no complications. Post-op CT scans demonstrated a reduced zygomatic bone with no impingement or dislocation of the coronoid process and reduction of the condylar subluxation.

The patient was discharged 24 h later with medical treatment for 1 week, soft diet, and physiotherapy for 6 weeks. The follow-up visit showed no signs of malunion, and the patient maintained good mouth opening.

### 2.1 Biomechanical rationale

This case underscores a rare mechanism of injury wherein a mandibular coronoid process becomes locked anterior to a fractured zygomatic complex, preventing mouth closure. The presumed biomechanics involve an interplay between condylar displacement^5^ and coronoid process movement.

We postulate that at the moment of impact, the patient's mouth was likely open, or the condyle was forcibly dislocated, allowing the coronoid process to escape out of the temporal fossa.^6^ When the patient attempted to close the mouth after the fall, the intact coronoid process collided with the displaced zygomatic arch fragment and became trapped in front of it, effectively wedging the jaw open.

This sequence also pushed the condyle out of the glenoid fossa (anterolateral TMJ subluxation). Notably, in our case (and the similar case by Nini et al.),^1^ the coronoid process did not fracture despite the severe force, likely due to its stout structure and the “give” provided by condylar subluxation^6^ and stretching of the attached temporalis muscle. (Coronoid fractures generally result from direct high-force impaction or powerful muscle contraction,^7^ and their absence here suggests the energy was dissipated through joint subluxation and bone displacement instead.)

## Sources of support/grants

None.

## Patient's consent for the photographs obtained

Yes.

## Ethical statement

The proposed techinique entitled - Atypical Coronoid Process Displacement in ZMC Trauma: Technical Considerations and Management Algorithm – A Technical Note: The surgical techinique is well within ethical norms and ethically justified. However, informed written consent will be taken from the patients participating in the present study. Informed consent will be taken.

## Funding

The proposed techinique entitled - Atypical Coronoid Process Displacement in ZMC Trauma: Technical Considerations and Management Algorithm – A Technical Note: received no sources of funding.

## Declaration of competing interest

The authors declare that they have no known competing financial interests or personal relationships that could have appeared to influence the work reported in this paper.
